# The Features of Foot Morphology and Intrinsic Foot Muscle Property in Adolescent Swimmers: An Ultrasound-Based Study

**DOI:** 10.5114/jhk/163148

**Published:** 2023-07-15

**Authors:** Kazuki Kaneda, Noriaki Maeda, Yasunari Ikuta, Tsubasa Tashiro, Shogo Tsutsumi, Satoshi Arima, Junpei Sasadai, Yuta Suzuki, Masanori Morikawa, Makoto Komiya, Nobuo Adachi, Yukio Urabe

**Affiliations:** 1 Department of Sports Rehabilitation, Graduate School of Biomedical and Health Sciences, Hiroshima University, Hiroshima, Japan.; 2 Department of Orthopedic Surgery, Graduate School of Biomedical and Health Sciences, Hiroshima University, Hiroshima, Japan.; 3 Sports Medical Center, Hiroshima University Hospital, Hiroshima, Japan.; 4 Sports Medical Center, Japan Institute of Sports Sciences, Japan Sport Council, Tokyo, Japan.; 5 Department of Physical Therapy, Faculty of Rehabilitation, Kyusyu Nutrition Welfare University, Fukuoka, Japan.; 6 Department of Preventive Gerontology, Center for Gerontology and Social Science, National Center for Geriatrics and Gerontology, Aichi, Japan.

**Keywords:** adolescent, athletes, flexibility, kinesiology, ultrasonography

## Abstract

This cross-sectional study aimed to investigate the relationship between foot shape and intrinsic foot muscles (IFMs) in adolescent swimmers compared with other athletes. Foot morphology of swimmers was compared with other athletes of comparable age and a competition level (n = 32 [64 feet]; a total of 64 feet; 128 feet in two groups). Foot morphology and variables of IFMs were measured using a three-dimensional foot scanner and an ultrasound imaging system, respectively. Multiple linear regression analysis with forced entry was performed to clarify the association of the thickness and the cross-sectional area (CSA) of IMFs with the navicular height in both sitting and standing positions. Navicular height in a standing position, the thickness of the abductor hallucis (AbH) and the flexor digitorum brevis (FDB), as well as the CSA were significantly lower in swimmers compared with other athletes (p < 0.05). A larger CSA for the flexor hallucis brevis (FHB) was observed in swimmers than in other athletes (p < 0.01). The navicular height of swimmers in sitting and standing positions was positively correlated with the thickness and the CSA of the FHB and the FDB (p < 0.05). Multiple regression analysis showed that navicular height was associated with the CSA of the FDB in both sitting and standing positions (β = 0.395; p < 0.002, β = 0.201; p < 0.018). This study showed that the navicular height of elite adolescent swimmers was lower than that of other athletes. Furthermore, the swimmers’ high navicular height was associated with the CSA of the FDB, suggesting that the FDB might be more involved in the formation of the medial longitudinal arch than the AbH.

## Introduction

Competitive swimmers have more foot flexibility than other athletes due to the general laxity of their joints (Shimojo et al., 2019), and their excessively flexible feet are typically flat-footed regardless of the growth and development process (Wanivenhaus et al., 2012). Foot injuries in competitive swimmers generally occur during dry land resistance training (Wolf et al., 2009); however, previous studies have not explicitly discussed swimmers’ foot morphology. Therefore, it is important to investigate the details of the swimmers’ foot posture in terms of the perspective of injury prevention.

Flat feet were reported in the relationship between balance ability, foot muscle fatigue (Gray and Basmajian, 1968; Tsai et al., 2006), sports performance, and sports-related injury (Tourillon et al., 2019). The medial longitudinal arch (MLA) is considered an important variable with a consensus for measuring and evaluating foot morphology (Villarroya et al., 2009), and its development is necessary and indispensable in daily life and sporting activities. Recently, Kobayashi et al. (2020) identified lower navicular height in competitive swimmers than in other college female athletes using a three-dimensional (3D) foot scanner. The development of intrinsic foot muscles (IFMs) in response to loading aids MLA formation (Kelly et al., 2014). On the other hand, structural factors that lead to a MLA decrease are related to the thickness of IFMs and the size of the cross-sectional area (CSA) (Angin et al., 2014). Swimmers have fewer opportunities for running and jumping on the ground than other athletes because they practice mainly in the water environment. There is a lot of research on swimmers based on their performance in the water; however, no research has investigated basic foot morphology, and furthermore, no comparison of adolescents with other competitive athletes has been made.

Therefore. this study aimed to investigate the relationship between the foot posture and IFMs in competitive adolescent swimmers compared with other age-matched athletes of a similar competitive level. We hypothesized that swimmers would exhibit flatfoot and insufficient development of IFMs compared to other athletes.

## Methods

### Participants

Thirty-two swimmers and 32 athletes from other sports (rugby, kendo, ice hockey, ballet, basketball, and volleyball) participated in the study. Of the other athletes included, rugby, kendo, ice hockey, and ballet dancers tended to have flat feet, while basketball and volleyball players tended to have relatively high foot arches. All participants were elite adolescent athletes registered by the prefectural sports association, with at least 5 year competitive experience. Inclusion criteria were (1) age between 12 and 18 years, (2) membership of a club team within the prefecture, and (3) designation as a certified athlete by the prefecture. The exclusion criteria were: (1) current disease or (2) plantar fasciitis, ligamentous injuries, or orthopedic injuries to the lower limb.

The study was executed in accordance with the Declaration of Helsinki and was approved by the Epidemiology Ethics Committee of the Hiroshima University (approval number: E-2090). All participants provided informed consent to participate in the study. In the case of minors, they participated in the study with the consent of their parents or legal guardians.

### Measures

#### Measurement of the Foot Morphology using 3D Foot Scanning

**Figure 1:**
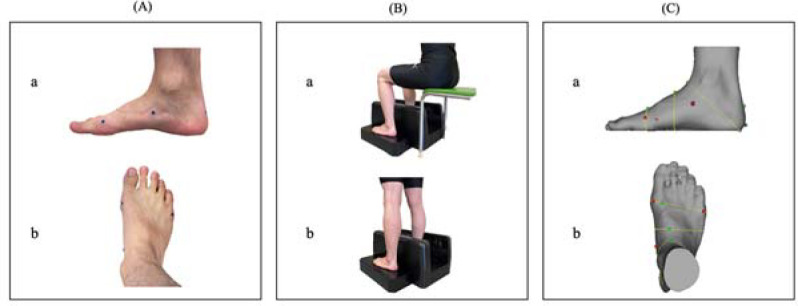
(A) Position of the marker: a) sagittal plane, b) horizontal plane. (B) Participant positioned in the INFOOT2 digitizer: a) sitting position, b) standing position. (C) Scanned image: a) sagittal plane, b) horizontal plane.

Foot morphology was measured using a 3D foot scanner (INFOOT2 USB scanning system, IFU2-S-01, I-ware Laboratory, Ltd., Osaka, Japan). This scanner has shown substantial to near-perfect inter- and intra-rater reliability and has established validity compared to clinical caliper data and radiographic measurements (De Mits et al., 2010). The system measures the 3D shape of the feet using eight cameras to capture the projected lines of line laser light emitted from four red lasers in the form of a plane. Then, the accumulated CSA is cut in a circle using the optical cutting method. Therefore, anatomical dimensions can be accurately obtained using a non-contact 3D measuring machine (De Mits et al., 2011; Lee et al., 2014). Before scanning, a technician with 5 years of experience attached special markers to the four landmark positions of each participant’s foot: (1) the most medial point of the first metatarsal head, (2) the most lateral point of the fifth metatarsal head; (3) the most protruded point of the calcaneus; and (4) the most protruded point of the navicular tuberosity (Hill et al., 2017). Participants were instructed to sit up straight and look straight ahead, with their weight distributed equally between both feet. Following a scan of both feet in the seated position, the same measurements were obtained in the standing position. Figure 1 shows the details of the measurement. The foot scanning system could automatically detect landmark locations, calculate foot measurements, and accurately measure sitting and standing foot length, foot width, and navicular height. The analysis was performed using software (Footprint Measurement; Measure) that came with the device.

#### Evaluation of Muscle Thickness and CSA

Morphologies of IMFs (thickness and CSA) were observed using ultrasonography (HI VISION Avius; Hitachi Aloka Medical, Tokyo, Japan). An 8-MHz linear array probe was used to obtain an ultrasound image. The ultrasound measurements were evaluated by a technician with 5 years of experience.

**Figure 2:**
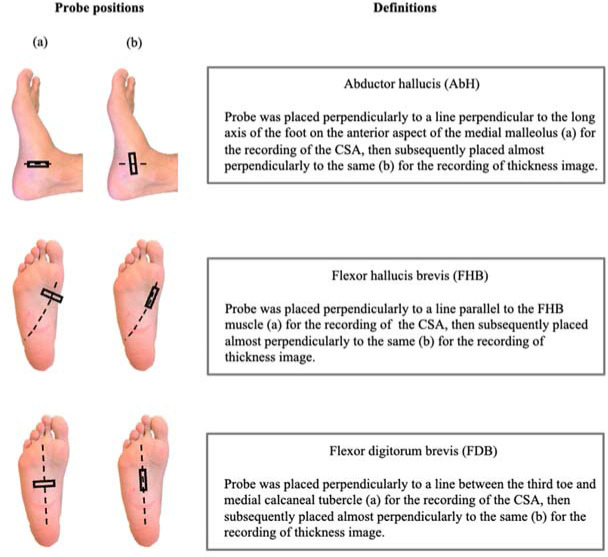
The probe’s position in the assessment of selected tissue thickness and the CSA. Abbreviations: AbH, abductor hallucis; FHB, flexor hallucis brevis; FDB, flexor digitorum brevis.

Measurements were performed in the prone position, at 90 degrees of knee flexion, and in a neutral ankle position. The following variables were measured: thickness and the CSA of the abductor hallucis (AbH), flexor hallucis brevis (FHB), and flexor digitorum brevis (FDB). Details of the probe positions and definitions are provided in Figure 2. The probe position was marked on the skin with semi-permanent ink. The probe was placed on the anterior side of the medial malleolus along a line perpendicular to the long axis of the foot’s longitudinal arch, and it recorded the CSA image of the AbH. Afterwards, it was placed approximately perpendicularly to the same line to record the thickness image. Furthermore, the probe was placed perpendicularly to a line parallel to the FHB to record its CSA image, and was subsequently placed along the same line to record the thickness image. Finally, the probe was placed along a line between the third toe and the medial calcaneal tubercle to record the CSA image of the FDB, and was subsequently placed along the same line to record the thickness image (Taş et al., 2020). These measurement methods have been previously reported by our research group (Maeda et al., 2021; Morikawa et al., 2021), and they are reliable in assessing the IFMs thickness and the CSA (Crofts et al., 2014).

### Design and Procedures

This cross-sectional study included swimmers and athletes of other sports to investigate the morphology of their feet and the IFM characteristics of elite adolescent swimmers. The study was conducted in a laboratory at the Hiroshima University. A technician with more than 5 years of experience carried out the procedures.

### Statistical Analysis

Data were analyzed using the SPSS version 27.0 for Mac (IBM Japan Co., Ltd., Tokyo, Japan). The Shapiro-Wilk test was used to test the normality of the data distribution. Normally and non-normally distributed variables are presented as mean ± standard deviation (SD). After normality was confirmed, the demographics, foot shape, foot intrinsic muscle thickness and the CSA were compared between elite adolescent swimmers and other athletes using the unpaired *t*-test if normality was present, and the Wilcoxon rank-sum test if not. Depending on data distribution, correlation coefficients were calculated using the Pearson's product-moment correlation coefficient test or the Spearman correlation coefficient test. They were used to estimate the relationships between thickness and CSA values of the assessed tissue (AbH, FDB, and FHB) and foot shape due to postural differences. Multiple linear regression analysis with forced entry was performed to examine the association of the assessed tissue thickness, the CSA, and navicular height by postural differences among swimmers. The navicular height of swimmers was the dependent variable, and the thicknesses of AbH, FDB, FHB, and the CSA were factors. To avoid overfitting due to a small sample size, we used variables with correlation coefficients of <0.10 between selected tissue morphology as objective variables. Statistical significance was set at *p* < 0.05.

In this study, a post hoc power analysis was conducted to confirm that data were sufficiently powerful. A post hoc power analysis is important for the reliability of the data (Faul et al., 2009). The analysis procedure required the population “Effect size f2,” the “α err prob,” the “Total sample size,” and the “Number of predictors” in the regression model as input parameters to obtain the power of F test (“Power [1 err prob]”).

## Results

The demographic data of swimmers and other athletes are shown in Table 1. Navicular height in a standing position, the thickness of AbH and FDB, and the CSA were significantly lower in swimmers than in other athletes (*p* < 0.05). The results of the navicular drop test and the CSA of FHB were significantly higher in swimmers than in other athletes (*p* < 0.01).

**Table 1:** Demographic and foot shape characteristics between swimmers and other athletes.

Wong, Y. S. (2007). Influence of the abductor hallucis muscle on the medial arch of the foot: a kinematic and anatomical cadaver study. *Foot and Ankle International, 28*(5), 617–620. https://doi.org/10.3113/FAI.2007.0617	Swimmers (n = 32, 64 foot)	Other athletes (n = 32, 64 foot)	*p*-value
Gender (Male: Female)	20:12	20:12	
Age	15.9 ± 2.4	15.3 ± 2.4	0.361
Body Height (m)	1.63 ± 0.07	1.64 ± 0.07	0.841
Body Weight (kg)	54.5 ± 7.5	54.4 ± 9.2	0.979
BMI (kg/m^2^)	20.4 ± 1.7	20.3 ± 2.5	0.858
Foot shape (mm)			
Foot size in the sitting position	242.9 ± 11.5	241.4 ± 14.5	0.515
Foot size in the standing position	243.6 ± 11.8	244.7 ± 14.7	0.633
Foot width in the sitting position	96.9 ± 5.8	97.3 ± 7.1	0.693
Foot width in the standing position	97.0 ± 5.8	100.0 ± 6.9	0.051
Navicular height in the sitting position	41.0 ± 6.2	43.3 ± 7.6	0.061
Navicular height in the standing position	35.6 ± 6.4	39.4 ± 7.1	**0.002**
Navicular Drop test	5.4 ± 2.7	4.0 ± 2.9	**0.006**
Thickness of selected tissues (mm)			
Abductor hallucis	11.0 ± 1.9	12.1 ± 2.3	**0.005**
Flexor hallucis brevis	11.7 ± 2.0	11.4 ± 1.7	0.208
Flexor digitorum brevis	7.8 ± 1.3	8.3 ± 1.3	**0.002**
Cross-sectional area (mm^2^)			
Abductor hallucis	237.1 ± 56.0	256.5 ± 66.8	**0.040**
Flexor hallucis brevis	251.5 ± 47.3	228.7 ± 34.8	**0.003**
Flexor digitorum brevis	191.5 ± 53.0	221.3 ± 51.4	**0.002**

Data are shown as mean ± standard deviation, BMI: Body Mass Index, p < 0.05.

The bolded values were set at p < 0.05.

The navicular height of swimmers in the sitting and standing positions was positively correlated with the thickness of the FHB and FDB and the CSA of the FHB and FDB (Table 2). In addition, the navicular drop test results were positively correlated with the CSA of the AbH and the thickness of the AbH.

**Table 2:** Relationship between navicular height by posture difference with muscle thickness and the muscle cross-sectional area.

Variables	Swimmers						Other athletes					
	Navicular height in the sitting position		Navicular height in the standing position		Navicular Drop		Navicular height in the sitting position		Navicular height in the standing position		Navicular Drop	
	r	*p*	r	*p*	r	*p*	r	*p*	r	*p*	r	*p*
Thickness (mm)												
AbH	−0.072	0.574	−0.199	0.114	0.331**	<0.007	−0.058	0.648	−0.096	0.451	0.095	0.455
FHB	0.295*	<0.018	0.276*	<0.027	−0.136	0.285	0.044	0.730	0.053	0.680	−0.030	0.815
FDB	0.301*	<0.016	0.284*	<0.023	0.079	0.709	0.099	0.436	0.075	0.521	0.031	0.811
CSA (mm^2^)												
AbH	0.052	0.686	−0.072	0.573	0.320**	<0.010	−0.036	0.779	−0.099	0.437	0.137	0.280
FHB	0.416**	<0.001	0.284*	<0.023	0.048	0.709	0.162	0.200	0.082	0.521	0.275*	<0.028
FDB	0.400**	<0.001	0.296*	<0.017	0.211	0.094	0.076	0.549	−0.026	0.836	0.207	0.100

* : p < 0.05, **: p < 0.01. Each variable was tested by the Pearson correlation coefficient. Abbreviation: CSA: Cross-sectional area. AbH: abductor hallucis, FHB: flexor hallucis brevis, FDB: flexor digitorum brevis.

Table 3 summarizes the results of the multiple regression analysis of the association between the IFM morphology and the navicular height in swimmers. In both sitting and standing positions, the navicular height was associated with the CSA of the FDB. In the post hoc power analysis, the multivariate regression model for the association between ultrasonographic assessment items and the navicular height in swimmers demonstrated sufficient power (1 err prob = 0.998).

**Table 3:** Multiple regression analysis of navicular height and muscle cross-sectional area by postural differences among swimmers.

Variables	Navicular height in the sitting position						Navicular height in the standing position				
	β	95% CI interval		*p*-value		β		95% CI interval		*p*-value	
		Lower	Upper					Lower	Upper		
CSA of AbH(mm^2^)	−0.114	−0.039	0.014	0.338		−0.217		−0.053	0.003	0.083	
CSA of FHB (mm^2^)	0.238	0.000	0.063	0.050		0.358		−0.001	0.067	0.055	
CSA of FDB (mm^2^)	0.395	0.018	0.075	<0.002		0.201		0.007	0.067	<0.018	

Explanatory variables were the CSA of abductor hallucis, flexor hallucis brevis, and flexor digitorum brevis.

β: standardized partial regression coefficient, Abbreviation: CSA: Cross-sectional area,

AbH: abductor hallucis, FHB: flexor hallucis brevis, FDB: flexor digitorum brevis.

## Discussion

This is the first study to clarify the relationship between foot morphology and IFMs in elite adolescent swimmers. This study revealed that navicular height in a standing position was significantly lower in swimmers than other age-matched athletes of a comparable competitive level. Navicular height moderately correlated with the CSA of the FHB and the FDB in swimmers. Furthermore, the CSA of the FHB was significantly larger in swimmers than in other athletes, yet multiple regression analysis showed that the FDB effected navicular height in swimmers.

Elite swimmers spend 2–5 hours swimming approximately 5,000 to 7,000 m in a single practice session (Trinidad et al., 2021). Joint laxity increases with age due to decreased loading opportunities caused by frequent daily practice in swimmers (Khodaee et al., 2015; Jansson et al., 2005). The MLA of the foot is not present at birth and gradually develops over a period of approximately 10 years to become the natural arch formation process (Pita-Fernandez et al., 2017). In general, the incidence of flatfoot is approximately 20% in adolescents and decreases with aging (Bhoir et al., 2014) due to the development of IFMs and the attainment of foot stiffness by loading response (Kelly et al., 2014). The foot morphology of other athletes who participated in this study was similar to the results of previous studies (Kobayashi et al., 2020; Rogati et al., 2019). Although a diverse group of athletes participated in this study, it was probable that they would obtain a common consensus as load-bearing athletes in sports. Meanwhile, adolescent swimmers had significantly lower navicular height in a standing positions than other athletes, and their muscle thickness and CSA of the AbH and the FDB were smaller. These findings might have been due to their competitive characteristics and less loading opportunity. In a previous study, the competitive swimmers’ foot was not discussed as much as the swimming shoulder, lower back, and knee (Hill et al., 2022). However, most foot injuries occur during dry land resistance training and warm-ups in competitive swimmers, and ankle sprain and instability are considered key issues due to the chronic symptoms (Wolf et al., 2009). Therefore, from our results, the low MLA in swimmers due to insufficient development of the IFMs could be a point of note.

Moreover, navicular height in the standing position was lower in adolescent swimmers than in other athletes. Among IFMs, the AbH mainly contributes to MLA formation (Kelly et al., 2014); thus, the deterioration of AbH function leads to a decrease in MLA height (Wong, 2007). The AbH is the largest of the IFMs that causes flexion and supination of the first metatarsal and inversion of the calcaneus, thereby contributing to the elevation of the MLA and compensating for foot stiffness (Fiolkowski et al., 2003).

In contrast, the FDB is involved in the response to loading and postural balance, similarly to the plantar fascia, and is less associated with the MLA formation than the AbH (Tosovic et al., 2012). However, the FDB was the variable that correlated best with navicular bone height in swimmers; it was not the FHB, which was greater than in other athletes. Unlike other IFMs, the FHB is a flexor of the intermetatarsophalangeal joint because it attaches from the cuboideum bone and lcuneiforme laterale bone to the base of the proximal phalanges (Latey et al., 2014). Competitive swimmers typically use a flutter kick motion that requires excessive ankle plantar flexion and forefoot flexion (McCullough et al., 2009). Therefore, it is possible that the FHB, which is involved in metatarsophalangeal joint flexion, is more active than the other IFM, and develops muscle thickness and the CSA. Although the FHB is second only to the AbH in importance for MLA formation (Latey et al., 2014), swimmers gain less foot stability due to fewer loading opportunities, and even with a larger FHB, swimmers with flexible feet are unable to maintain MLA. Thus, the existence of the FDB and not the AbH or FHB might result in MLA formation in adolescent swimmers.

This study has several limitations. First, it was a cross-sectional study; therefore, whether previous swimming experience or athletic history was related to the results was not sufficiently determined. Second, the results were for mixed gender athletes, and there was no comparison between genders. Nevertheless, this study allowed us to better understand the cross-sectional foot characteristics of elite adolescent swimmers.

In conclusion, this study revealed that the navicular height of elite adolescent swimmers was lower than that of other athletes of the same age bracket and competitive level. In addition, a higher navicular height in swimmers was associated with the CSA of the FDB. The FDB may have a greater involvement in compensating for arch formation than the AbH in swimmers. These results provide fundamental data that will aid in understanding of foot characteristics among elite adolescent swimmers.
